# The global burden of chronic hepatitis B virus infection: comparison of country-level prevalence estimates from four research groups

**DOI:** 10.1093/ije/dyaa253

**Published:** 2020-12-27

**Authors:** Nora Schmit, Shevanthi Nayagam, Mark R Thursz, Timothy B Hallett

**Affiliations:** 1 MRC Centre for Global Infectious Disease Analysis, Department of Infectious Disease Epidemiology, Faculty of Medicine, Imperial College London, London, UK; 2 Division of Digestive Diseases, Department of Metabolism, Digestion and Reproduction, Imperial College London, London, UK

**Keywords:** Hepatitis B, viral-hepatitis elimination, prevalence, disease burden, infectious diseases, modelling, indicator, monitoring, sub-Saharan Africa

## Abstract

**Background:**

Progress towards viral hepatitis elimination goals relies on accurate estimates of chronic hepatitis B virus (HBV)-infection prevalence. We compared existing sources of country-level estimates from 2013 to 2017 to investigate the extent and underlying drivers of differences between them.

**Methods:**

The four commonly cited sources of global-prevalence estimates, i.e. the Institute for Health Metrics and Evaluation, Schweitzer *et al.*, the World Health Organization (WHO) and the CDA Foundation, were compared by calculating pairwise differences between sets of estimates and assessing their within-country variation. Differences in underlying empirical data and modelling methods were investigated as contributors to differences in sub-Saharan African estimates.

**Results:**

The four sets of estimates across all ages were comparable overall and agreed on the global distribution of HBV burden. The WHO and the CDA produced the most similar estimates, differing by a median of 0.8 percentage points. Larger discrepancies were seen in estimates of prevalence in children under 5 years of age and in sub-Saharan African countries, where the median pairwise differences were 2.7 percentage and 2.4 percentage points for all-age prevalence and in children, respectively. Recency and representativeness of included data, and different modelling assumptions of the age distribution of HBV burden, seemed to contribute to these differences.

**Conclusion:**

Current prevalence estimates, particularly those from the WHO and the CDA based on more recent empirical data, provide a useful resource to assess the population-level burden of chronic HBV-infection. However, further seroprevalence data in young children are needed particularly in sub-Saharan Africa. This is a priority, as monitoring progress towards elimination depends on improved knowledge of prevalence in this age group.

**Key Messages**

- Accurate estimates of chronic hepatitis B virus (HBV)-infection prevalence across all ages and in children <5 years of age are essential to assess country-level burden and monitor progress towards viral hepatitis elimination.
- Despite differences in methodology, we found that the four existing sources of country-level HBV prevalence estimates across all ages were similar in most countries.
- There were larger discrepancies in estimates in children <5 years of age and in sub-Saharan African countries, which were in part driven by differences in the recency and representativeness of included data and different modelling assumptions of the age distribution of HBV burden.
- Available estimates allow assessment of national population-level HBV burden, but seroprevalence data in young children in sub-Saharan Africa were identified as a priority for further data collection to inform intervention priorities.

## Introduction

Chronic infection with the hepatitis B virus (HBV) is a major cause of chronic liver-disease and remains endemic in many countries despite the worldwide implementation of vaccination. The World Health Assembly has adopted the goal of eliminating viral hepatitis as a major public health threat by 2030, calling for a 90% reduction in new infections and a 65% reduction in mortality.^1^ Establishing the baseline HBV burden and monitoring progress towards these targets on the global, regional and country levels is important to prioritize health-resource allocation, advocate for action and investment, and evaluate the impact of interventions by international organizations, funding bodies and governments.^2^ This is of particular priority in sub-Saharan Africa, where vaccine coverage has remained below the WHO targets,^1^ and many countries are only now beginning to develop national strategic plans for elimination.^3^^,^^4^

Quantification of chronic HBV burden relies on seroprevalence studies for the hepatitis B surface antigen (HBsAg) in the general population.^5^ Whereas HBsAg prevalence in adults gives an indication of the scope of the epidemic and potential liver-disease burden, an accurate estimate of the prevalence in children aged 5 years is especially important because it is a proxy indicator for the cumulative incidence of chronic infection and reflects the impact of vaccination programmes.^6^^,^^7^ However, population-based nationally representative HBsAg seroprevalence measurements are lacking in many countries^8^ and a particular paucity of reliable prevalence data has been reported in sub-Saharan Africa.^9^ Four research groups have attempted to address these data gaps by synthesizing empirical measurements from various sources to calculate improved and comparable estimates of chronic HBV prevalence worldwide.^10^ In 2015, Schweitzer *et al.* published the first global systematic review and pooled analysis of country-level HBsAg seroprevalence.^5^ Recent modelled estimates have been produced by the Institute for Health Metrics and Evaluation (IHME) as part of the Global Burden of Disease (GBD) study,^11^ the World Health Organization (WHO) in collaboration with the London School of Hygiene and Tropical Medicine to set the baseline for elimination targets^1^^,^^12^ and the non-profit CDA Foundation’s Polaris Observatory.^13^ Estimates of the current global chronic HBV prevalence from these four research groups range from 3.5% to 5.6% across all ages, and from 1.3% to 3.4% in children <5 years of age. However, it is not known to what degree these sets of estimates differ at the country level. Additionally, although the CDA found that sub-Saharan African countries constituted the largest sources of uncertainty in their global-prevalence estimates, the reasons for this were not further investigated.^13^

Previous analyses have shown that comparative studies of global health estimates can facilitate interpretation of the estimation process and its limitations, and highlight less reliable estimates and data gaps in specific countries.^14–17^ In this paper, we compare the four widely cited sets of country-level chronic HBV-infection prevalence estimates to determine how different they are, where differences arise and which methodological factors drive potential discrepancies in sub-Saharan African estimates.

## Methods

### Prevalence estimates

Country-level estimates of HBsAg prevalence across all ages were collated from the publications by Schweitzer *et al.*^5^ and the CDA/Polaris Observatory^13^ and online from the WHO HBsAg dashboard^12^ and the IHME Global Burden of Disease Results tool.^18^ We also downloaded age-specific estimates of prevalence in children <5 years of age, which were available from the CDA, the WHO and the IHME. Age-specific estimates that were reported as ‘<0.1%’ by the CDA were excluded.

Schweitzer reports pooled estimates for the 1965–2013 period. The WHO estimates, downloaded in November 2017, were from the March 2017 database update and refer to prevalence in 2015, and the CDA estimates describe prevalence in the year 2016. The IHME estimates for chronic HBV-infection prevalence in the 2017 GBD revision are contained within the ‘Cirrhosis and other chronic liver diseases due to hepatitis B’ cause group^11^ and estimates for the ‘All ages’ and ‘Under 5’ age groups were downloaded for 2017. The ‘rate’ metric for the ‘prevalence’ measure in the results tool refers to cases per 100 000 population and was converted into percent prevalence.

Information on input data sources by country was extracted for sub-Saharan African countries from the CDA publication and the WHO dashboard. Countries were grouped by region according to the GBD classification and further combined into broad world regions.

### Data analysis

All analyses were conducted using R statistical software on country-level point prevalence estimates. We described percent-prevalence estimates by research group and by country, analysed pairwise differences between sets of estimates and compared the within-country variation in estimates globally. We did not investigate differences in the reported uncertainty intervals, as their meaning and interpretation varied between the different sets.

#### Pairwise differences between sets of estimates

The magnitude of differences in point estimates between groups was assessed through pairwise comparisons of the four sets of estimates, on all countries within the pair unless indicated otherwise. Absolute percentage-point differences for each pair of country-specific prevalence estimates, referred to as *pairwise absolute differences*, were calculated because the absolute percentage prevalence signals the overall public health importance of high-burden countries. We also compared the *pairwise relative difference*, calculated as the pairwise absolute difference between two estimates divided by the mean of the two estimates, to identify differences that were independent of the absolute level of prevalence.

To investigate whether estimates in children <5 years old differ more between groups than estimates across all ages, we compared their respective median pairwise relative difference across the same countries and groups.

#### Within-country variation in estimates from the different groups

To assess where estimates of all-age prevalence and of prevalence in children <5 years old were most different to one another, we calculated the country-specific mean absolute deviation (MAD) of prevalence estimates from different sets as shown in Equation 1. The MAD across estimates was calculated for all countries covered by at least two groups.

$$\begin{array}{c} {\mathrm{MAD}}_{a,c}=\frac{\sum_{g}^{N}{|p}_{a,c,g}-\bar{p_{a,c}}|}{N}\;\;\left(1\right) \end{array}$$

where

${\mathrm{MAD}}_{a,c}=\;$ mean absolute deviation of prevalence estimates for age group a in country c

$p_{a,c,g}\;=\;$ prevalence estimate for age group a in country c from set g

$\bar{p_{a,c}}\;=\;$ mean of all prevalence estimates for age group a in country c

N =  total country- and age group-specific number of prevalence estimates

### Identifying reasons for differences in sub-Saharan African estimates

To identify reasons for the differences in sub-Saharan Africa between sets of estimates, we summarized the methodological differences and compared input data sources and the modelled age distribution of HBV burden.

First, we investigated how the magnitude of pairwise relative differences varies with the inclusion of recent empirical input data and studied the correlation between country-specific pairwise relative differences and the number of included empirical studies using Spearman’s rank correlation. The WHO dashboard was used as a reference to determine the recency and number of empirical seroprevalence studies in each country, as they reported this in an accessible format. A reliance on recent data was defined to mean that studies published after 1 January 2013 were included in the estimates. Second, we calculated the prevalence ratios of the country-specific estimate across all ages divided by the corresponding estimate in children <5 years of age and compared their distributions between the IHME, the WHO and the CDA.

## Results

### The global HBV burden

**Figure 1.**
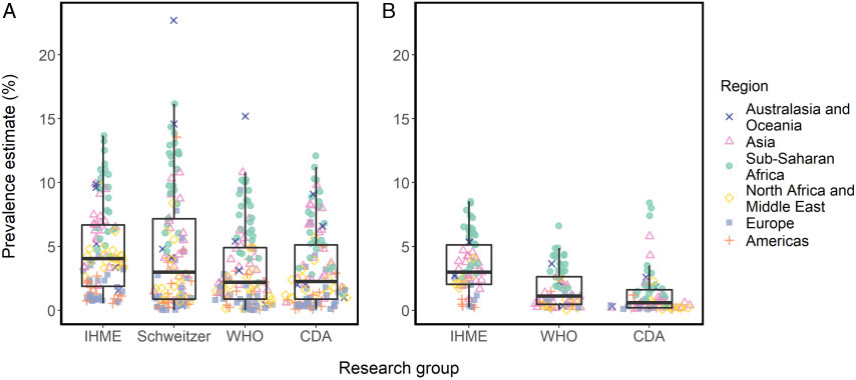
Distribution of country-level estimates of chronic HBV infection prevalence (A) across all ages and (B) in children under 5 years of age, from the Institute for Health Metrics and Evaluation (IHME), Schweitzer et al, the World Health Organization (WHO) and the CDA Foundation (CDA). Dots represent country-specific estimates spread according to the density distribution of the data, for the 112 (A) and 72 (B) countries covered by the four groups.

Recent national prevalence estimates were available from the IHME, Schweitzer, the WHO and the CDA for 195, 161, 194 and 120 countries, respectively. The four sets of estimates broadly agreed on the global distribution of HBV burden: the highest prevalence across all ages was concentrated in sub-Saharan Africa, Oceania and parts of Central, East and South East Asia, compared with low endemicity in countries in Western Europe and the Americas (Figure 1 and Supplementary Figure 1, available as Supplementary data at *IJE* online).

The median IHME estimate was 1.8 times higher than those from the WHO and the CDA when comparing prevalence estimates across all ages in the 112 countries covered by all four groups (Figure 1A). The median prevalence of the other sets of estimates were similar, but Schweitzer estimates were more variable than the other sets. Despite the IHME estimates being higher on average, pairwise comparisons with the other groups showed that large differences were confined to only a few countries. In half of the countries, estimates lay within 1.6 percentage points of each other [interquartile range (IQR) 0.6–3.0]. The median pairwise absolute difference between estimates from the WHO with Schweitzer and the WHO with the CDA was even lower, at 1.1 (IQR 0.3–2.8) and 0.8 (IQR 0.3–1.9) percentage points, respectively (Supplementary Table 1, available as Supplementary data at *IJE* online).

The same patterns were found for estimates of prevalence in children <5 years of age: the different sets of estimates agreed on the global distribution (Supplementary Figure 1, available as Supplementary data at *IJE* online) but the median IHME estimate was 2.7 and 5 times higher than those from the WHO and the CDA, respectively (Figure 1). However, discrepancies between groups were larger in age-specific estimates overall, with a median pairwise relative difference across all pairs and countries of 95% (IQR 50–138%) for estimates in children <5 years old compared with 39% (IQR 18–77%) for estimates across all ages.

**Figure 2.**
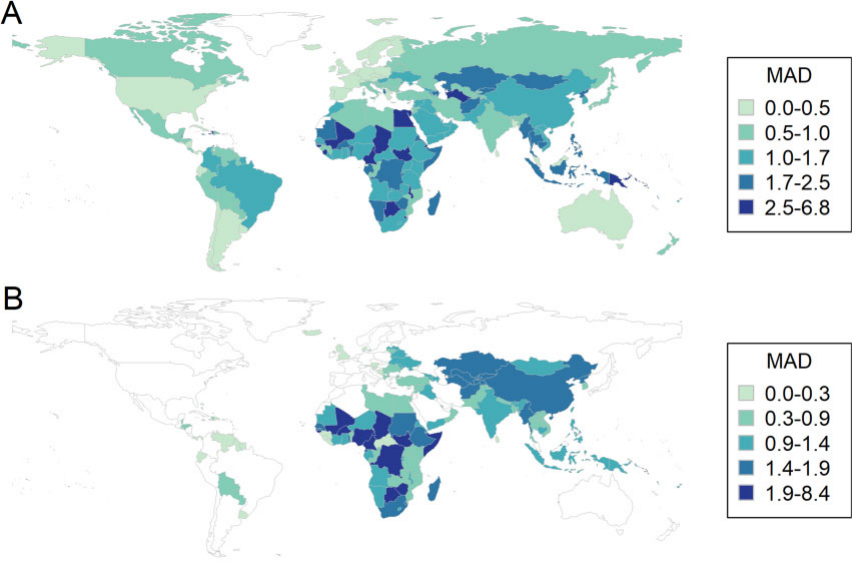
Within-country variation in estimates from different groups (A) for chronic HBV infection prevalence across all ages and (B) for chronic HBV infection prevalence in children under 5 years of age. Within-country variation is represented by the mean absolute deviation (MAD), and categories show the 25th, 50th, 75th and 90th percentile of MAD values. White shading represents countries where the MAD could not be calculated because less than two groups provided estimates.

Geographically, both national and age-specific estimates varied the most between groups in sub-Saharan African countries (Figure 2). The highest MAD was recorded in estimates in South Sudan, Swaziland and Sao Tome and Principe. This high variation in estimates in sub-Saharan African countries was particularly notable for prevalence in children <5 years old, where the MAD lay above 1.9 percentage points in 29% of 49 sub-Saharan African countries compared with 9% of 11 countries in Oceania and none of the modelled countries in Asia, North Africa and the Middle East, Europe or the Americas.

### Reasons for differences in sub-Saharan African estimates

The different methods used by the four research groups to identify, include and combine empirical measurements of HBsAg prevalence are summarized in Table 1. As it was the region with the largest discrepancies between sets of estimates, we focused on sub-Saharan African countries to investigate potential reasons for these differences. Sub-Saharan African estimates of all-age prevalence differed on average by 2.7 percentage points (IQR 1.3–4.3) and estimates in children <5 years old by 2.4 percentage points (IQR 1.3–3.5) across all pairs and countries, with those from the WHO and the CDA being most similar to each other (Supplementary Table 1 and Supplementary Figure 2, available as Supplementary data at *IJE* online).

**Table 1.** Overview of input data sources of hepatitis B surface antigen (HBsAg) seroprevalence and modelling methods underlying the four sets of prevalence estimates of chronic HBV infection from the Institute for Health Metrics and Evaluation (IHME), Schweitzer *et al.*, the World Health Organization (WHO) and the CDA Foundation (CDA)

	IHME	Schweitzer	WHO	CDA
Data				
HBsAg data sources	Peer-reviewed literature and other data (e.g. grey literature, Ministry of Health reports) suggested by collaborators	Peer-reviewed literature	Peer-reviewed literature and unpublished data suggested by Member States	Peer-reviewed literature and other data (e.g. grey literature, Ministry of Health reports) suggested by national experts
Literature search	Systematic review conducted for Global Burden of Disease study 2013	Systematic review from January 1965–October 2013 in Medline, Embase, CAB Abstracts (Global health), Popline, Web of Science	Schweitzer systematic review + extension from October 2013-March 2017 in Embase, PubMed, Global Index Medicus, Popline, Web of Science	Literature review from Jan 1960-March 2016 in PubMed and Embase
Included study populations	Not reported for HBV specifically	Included general population, blood donors, healthcare workers, pregnant women. Excluded high-risk population groups, e.g. migrants, prisoners, people who inject drugs	Included general population, blood donors, healthcare workers, pregnant women. Excluded high-risk population groups, e.g. migrants, refugees	Included general population, healthcare workers, pregnant women. Excluded non-representative populations, e.g. blood donors^a^, people who inject drugs, specific ethnic groups
Quality assessment	Not reported for HBV specifically	Assessed representativeness of study data	Assessed representativeness of study data	Quality scoring based on generalizability, sample size and recency (year)
Included HBsAg studies	420 site-years from 74 countries/subnational locations	1800 from 161 countries	2034 from 147 countries	One study each from 120 countries^a^
Modelling methods				
Use of data and modelling method	Meta-regression model with disease-specific natural history and hierarchical random effects on geography	Meta-analysis	Meta-regression with fixed-effect covariates and geospatial random effects	Dynamic deterministic Markov disease-progression model calibrated to the single highest-quality prevalence estimate for each country^a^
Model covariates	Infant vaccine coverage, non-disease-specific covariates (e.g. age, sex, location and socio-demographic index). Prevalence estimation also depends on cause-of-death model estimating hepatitis B mortality	None	Age (three categories), sex, study bias, three-dose vaccine coverage, birth-dose vaccine, study from pre- or post-vaccination period, study location, GDP per capita	Model populated with demographic, intervention coverage (including infant and birth-dose vaccine) and various epidemiological and natural history data
Extrapolation for missing data	Yes	No	Yes	Yes^b^
Reported country-level output	Chronic HBV-infection prevalence annually between 1990 and 2017, for various age groups and by sex (195 countries)	HBsAg prevalence in the general population pooled for the 1965–2013 period (161 countries)	HBsAg prevalence in the pre-vaccination period and in 2015, across all ages and in children <5 years of age (194 countries)	HBsAg prevalence in 2016, across all ages and in children aged <5 years (120 countries)
Reference	^10^, details in Supplementary Appendix 1—acute hepatitis B	^5^	^11^	^12^

a One study for point estimate in each country; further studies, including in blood donors, used for uncertainty interval.

b Extrapolated national estimates across all ages were only shown within endemicity categories on a map and therefore excluded from this analysis. Estimates in children based on extrapolated age patterns were included.

**Figure 3.**
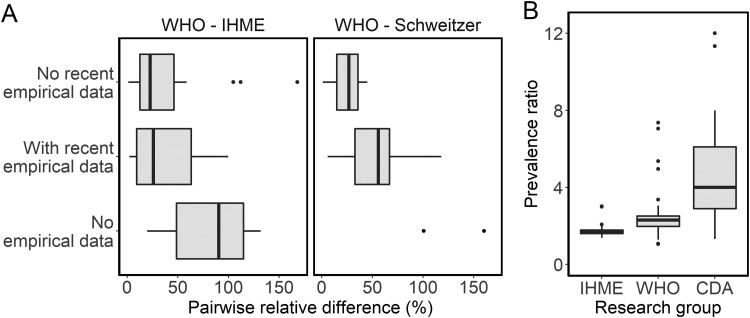
Factors contributing to differences in estimates of chronic HBV infection prevalence in sub-Saharan Africa. (A) shows the distribution of pairwise relative differences between estimates across all ages for different characteristics of the underlying empirical data, for (left) comparisons of estimates from the World Health Organization (WHO) with the Institute for Health Metrics and Evaluation (IHME), and (right) of WHO with Schweitzer estimates. (B) shows the prevalence ratio of estimates across all ages to estimates in children under 5 years of age by research group, which reflects the modelled age distribution of prevalence. The number of country-specific estimates represented in each category are: (A) 21 with no recent empirical data and 19 with recent empirical data for each comparison, 9 with no empirical data for WHO-IHME and 2 for WHO-Schweitzer, (B) 49 for IHME and WHO, 25 for the CDA Foundation (CDA). The 9 countries with no empirical data underlying the WHO estimate are Botswana, Chad, Comoros, Djibouti, Guinea-Bissau, Lesotho, Mauritius, Sao Tome and Principe, Swaziland.

For estimates of all-age prevalence, we found that extrapolation of estimates based on seroprevalence data from other countries contributed to differences between the WHO and the IHME, as some of the largest relative differences between these sets occurred in countries where no country-specific data were available (Figure 3A). Between the WHO and Schweitzer, relative differences were larger on average in countries where the WHO included a more recent seroprevalence study, published after the Schweitzer systematic review, than in countries without recent seroprevalence studies (Figure 3A). For the latter, estimates from these two groups were very similar in line with their overlap in data sources (Table 1). However, the availability of recent empirical data did not seem to affect differences between the WHO and the IHME, despite the IHME estimates also being based on an older systematic review (Figure 3A).

The literature reviews of the CDA and the WHO both included more recent data but, in contrast to the other groups, which pooled all available data meeting inclusion criteria, the CDA scored the identified studies for quality and only included the study deemed to be the most nationally representative in each country (Table 1). Although relative differences between the WHO and the CDA estimates were not correlated with the number of empirical seroprevalence studies underlying the WHO estimate in a given country (Spearman’s ρ = −0.07), some of the largest differences occurred in countries with the largest number of input studies, such as Nigeria (Supplementary Figure 2, available as Supplementary data at *IJE* online). In this and several other countries, the CDA prioritized more representative and recent studies over the range of seroprevalence surveys of varying scope and mostly conducted in specific population subgroups included by the WHO, although a single particularly high-quality seroprevalence study was not available in each country (Supplementary Table 2, available as Supplementary data at *IJE* online).

For estimates in children <5 years of age, differences were larger than for estimates of all-age prevalence overall, and varying patterns in the prevalence ratio of estimates across all ages to the corresponding age-specific estimate in children suggest different modelling approaches as a driver of these (Figure 3B). For the IHME and WHO estimates, the prevalence ratio was smaller and less variable across countries (range 1.4–2 and 1.5–5, respectively) than for the CDA, for which estimates across all ages were between 1.3 and 12 times higher than the corresponding age-specific estimate. As a result, the WHO estimates in children <5 years old were typically higher than those from the CDA (Supplementary Figure 2, available as Supplementary data at *IJE* online).

## Discussion

In this study, we compared the available country-specific estimates of chronic HBV prevalence across all ages and in children <5 years of age, generated by four different research groups. As the use of different modelled burden estimates in policy planning can potentially lead to very different conclusions about intervention priorities and resource allocation, we also elucidate the key drivers of differences and data gaps in HBV prevalence estimates. We found that the four sets of estimates agree on the overall global distribution of HBV burden, and that the WHO and the CDA produced remarkably similar estimates of all-age prevalence despite differences in their data sources and methodologies. However, estimates from the IHME were typically higher than those from other groups and the different groups produced disparate estimates of prevalence in children <5 years of age.

Current estimates highlight the high prevalence of chronic HBV infection remaining in sub-Saharan Africa and Oceania, although estimates in sub-Saharan African countries were among the most variable between the groups. Where estimates differ, this seems to be driven by a combination of methodological differences in identification and the use of underlying data sources, as well as different modelling assumptions. The comparison of sub-Saharan African estimates highlights the need for the inclusion of timely empirical seroprevalence data to produce reliable estimates of current burden. Schweitzer estimates may be more representative of historical seroprevalence in this region, as they are based on older data and do not account for the effects of vaccination. The addition of a vaccine covariate in the most recent revision of the IHME presented here seems to have reduced their global-prevalence estimates from the 2016 version (data not shown).^11^ A unique strength of their approach, involving a wide range of data to provide a comprehensive picture of disease burden including HBV-related mortality, may explain their higher estimates.^19^ However, the low quality of liver-disease mortality data across much of sub-Saharan Africa should be kept in mind when interpreting these estimates.^20^

Different perspectives taken by the CDA and the WHO on the available data illustrate the trade-off between including fewer datapoints and potentially introducing bias with less representative studies. The CDA method of prioritizing the highest-quality seroprevalence study may be preferable for countries with a range of data of varying quality and extensive consultation with local experts also allowed them to identify recent studies ahead of their publication in peer-reviewed journals. Conversely, the meta-analytic method used by the WHO made better use of the available information in countries where no representative study was available. It also enabled burden estimation for a larger number of countries, such as in South Africa, Namibia and the Democratic Republic of Congo, where the CDA found no suitable data to model prevalence according to their criteria, as available studies were conducted only in children, blood donors or specific ethnic groups.^13^ This trade-off highlights the utility of these models in complementing data-collection efforts to make an initial assessment of the extent of the hepatitis B epidemic, without replacing the need for empirical data in assessing national-level HBV burden and requirements for further interventions in the era of elimination.

In contrast to comparable prevalence estimates across all ages, the larger discrepancies in estimates in children <5 years old show that there remains considerable uncertainty about HBV burden in young children, particularly in sub-Saharan Africa. These differences seem to be driven by a lack of empirical data and a systematic difference in the modelling assumptions of the distribution of burden by age. The higher variation in the age-specific prevalence patterns across countries in the CDA estimates could be a result of their use of a dynamic mechanistic mathematical model, which may better account for the interplay between the effect of vaccination on HBV incidence and the age-dependent nature of the development of chronic infection than the statistical models used by the IHME and the WHO.^21^^,^^22^

Given the respective strengths of the different modelling approaches, and the most appropriate method likely depending on the available data, consultation of all available estimates or the underlying data sources may be preferable to assess country-specific data gaps. Future estimation efforts could involve the pooling of different methodological aspects or an ensemble average of models to combine the different perspectives, as has been applied in other examples of disease-burden estimation.^23^ However, our findings suggest that improvement of chronic HBV-infection prevalence estimates should primarily focus on the inclusion of robust and generalizable HBsAg seroprevalence studies. The results especially highlight the need to prioritize the collection of high-quality seroprevalence data in young children in sub-Saharan Africa to inform further prevention needs. Since modelled prevalence estimates in children <5 years of age are the only available evidence to assess reductions in chronic HBV incidence over time in some countries,^6^ the observed discrepancies could lead to confusion about progress towards elimination targets for local and international stakeholders.^24^ Additional data collection is also needed to clarify the burden in countries with no existing seroprevalence studies, since the different modelling methods produced highly discrepant extrapolated estimates. This could for example be facilitated by integrating HBsAg testing within existing national surveys like Demographic and Health Surveys.^25^^,^^26^

This study has some limitations. First, the comparison involved estimates for different years, which were pooled across the 1965–2013 period for Schweitzer and 2015–2017 for the other groups. Whereas this is unlikely to have major effects on estimates of all-age prevalence, differences in age-specific estimates may be sensitive to increasing effects of vaccination over time. Second, we only investigated the reasons for differences between the sets of estimates in sub-Saharan African countries due to their larger discrepancies but these factors may not be generalizable to other regions because of different epidemiology, vaccination history and research efforts. Third, our investigation of the drivers of differences was limited to the information on input data sources provided by the four groups and some assumptions were not clear, e.g. which estimates of prevalence in children <5 years of age were based on country-specific empirical data from that age group. Sharing of the literature reviews and extracted information in a more accessible format would facilitate further analyses and could also avoid repeated efforts to collate published data in the future.

## Conclusion

Despite differences in the use of data and modelling assumptions, the four research groups generated broadly similar estimates of current HBV prevalence. Available modelled estimates across all ages, particularly those from the WHO and the CDA based on more recent empirical data, allow prevalence to be compared globally and the national population-level HBV burden to be assessed. However, there was less agreement on country-specific estimates of prevalence in children <5 years of age, suggesting a need for further data collection in this age group, particularly in sub-Saharan Africa. Future estimation should focus on the inclusion of timely population-based seroprevalence data and could involve a combination of modelling approaches from the different groups.

## Supplementary data

Supplementary data are available at *IJE* online.

## Author contributions

N.S., S.N. and T.B.H. conceived of the study. N.S. conducted the analysis and wrote the manuscript. S.N., M.R.T. and T.B.H. provided guidance on the analysis and interpretation. S.N., M.R.T. and T.B.H. reviewed and revised the manuscript and approved the final version.

## Funding

This work was supported by the Imperial College Medical Research Council Doctoral Training Partnership (to N.S.); joint-centre funding from the UK Medical Research Council and Department for International Development (DFID) under the MRC/DFID Concordat agreement (grant number MR/R015600/1 to S.N. and T.B.H.); and funding from the National Institute for Health Research-Imperial Biomedical Research Centre (to S.N. and M.R.T.).

## Supplementary Material

dyaa253_Supplementary_Data
