# Supplementary material for: The global burden of chronic hepatitis B virus infection: comparison of country-level prevalence estimates from four research groups
Source: Int J Epidemiol. 2020 Dec 27;50(2):560–9. doi: 10.1093/ije/dyaa253 (PMC8128471; doi:10.1093/ije/dyaa253)
Supplement: dyaa253_Supplementary_Data [file dyaa253_supplementary_data.pdf]

Supplementary Data

A

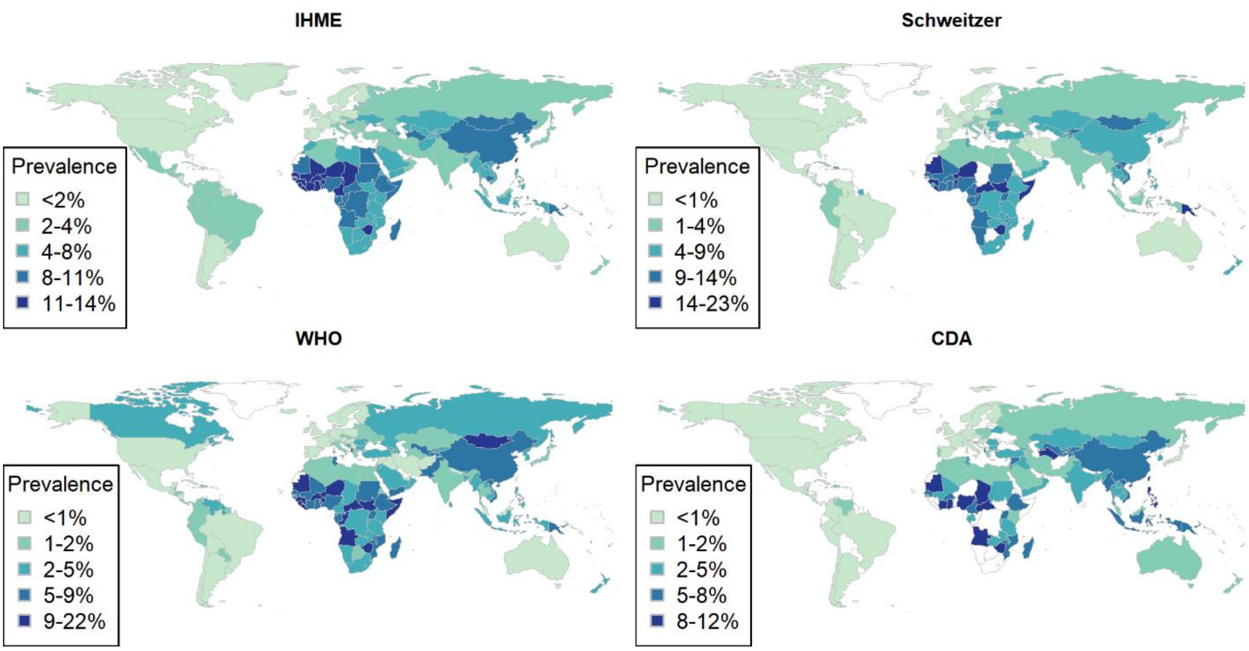

B

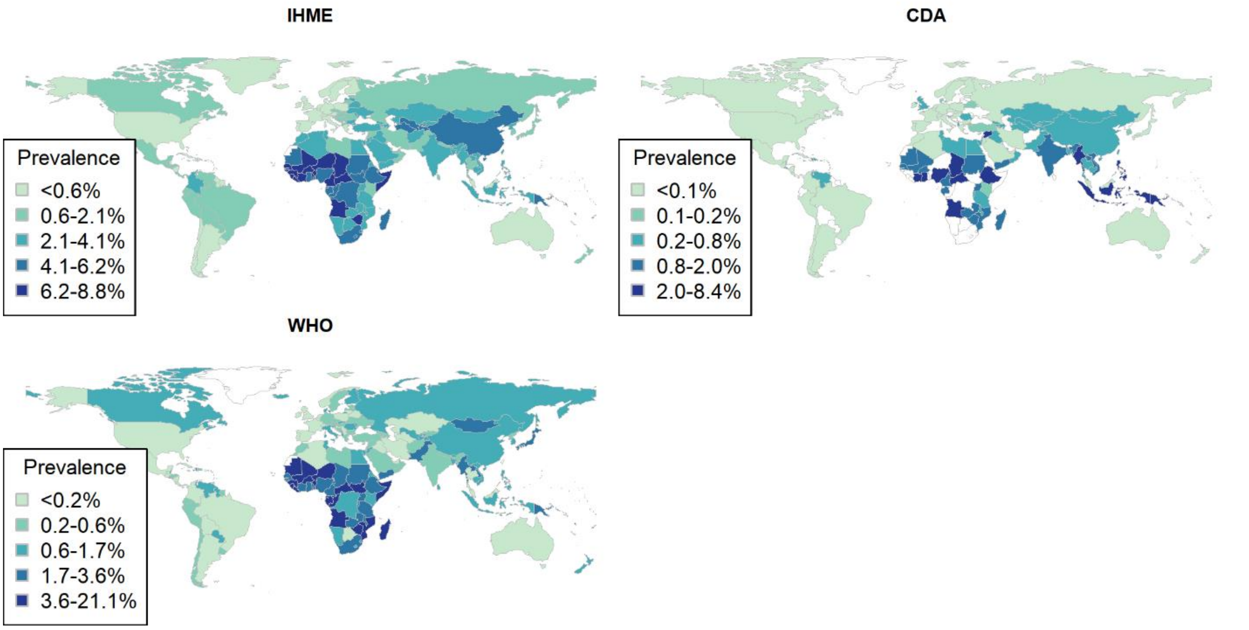

Supplementary Figure S1. Country-level estimates of chronic hepatitis B virus infection prevalence (A) across all ages and (B) in children under 5 years of age, from the Institute for Health Metrics and Evaluation (IHME), Schweitzer *et al*, the World Health Organization (WHO) and the CDA Foundation (CDA). Categories represent the 25th, 50th, 75th and 90th percentile of prevalence estimates for each set. CDA estimates reported as “<0.1%” are also shown in (B).

Supplementary Table S1. Pairwise absolute differences in estimates of chronic hepatitis B virus infection prevalence across all ages, from the Institute for Health Metrics and Evaluation (IHME), Schweitzer *et al*, the World Health Organization (WHO) and the CDA Foundation (CDA). Differences are shown for all estimates globally and for the subset of estimates in sub-Saharan African countries.

	All estimates (global)			Sub-Saharan African estimates		
**Pairwise comparison**	**Median (IQR[a])**	**Range**	***n*[b]**	**Median (IQR[a])**	**Range**	***n*[b]**
**IHME-CDA**	1.7 (0.6-3.0)	0.0-8.8	119	2.4 (1.9-3.0)	0.6-8.5	25
**IHME-Schweitzer**	1.7 (0.7-3.3)	0.0-15.4	155	2.8 (1.0-4.1)	0.0-15.4	42
**IHME-WHO**	1.4 (0.6-3.0)	0.0-15.2	186	2.4 (1.0-4.5)	0.1-15.2	49
**Schweitzer-CDA**	1.0 (0.5-3.0)	0.0-13.6	112	3.5 (2.1-6.1)	0.5-9.0	24
**Schweitzer-WHO**	1.1 (0.3-2.8)	0.0-16.9	158	2.9 (2.0-4.3)	0.1-16.9	42
**WHO-CDA**	0.8 (0.3-1.9)	0.0-7.7	118	1.9 (0.9-3.3)	0.1-5.7	25
**All pairs**	1.3 (0.5-2.9)	0.0-16.9		2.7 (1.3-4.3)	0.0-16.9	

[a] interquartile range

[b] number of countries in comparison

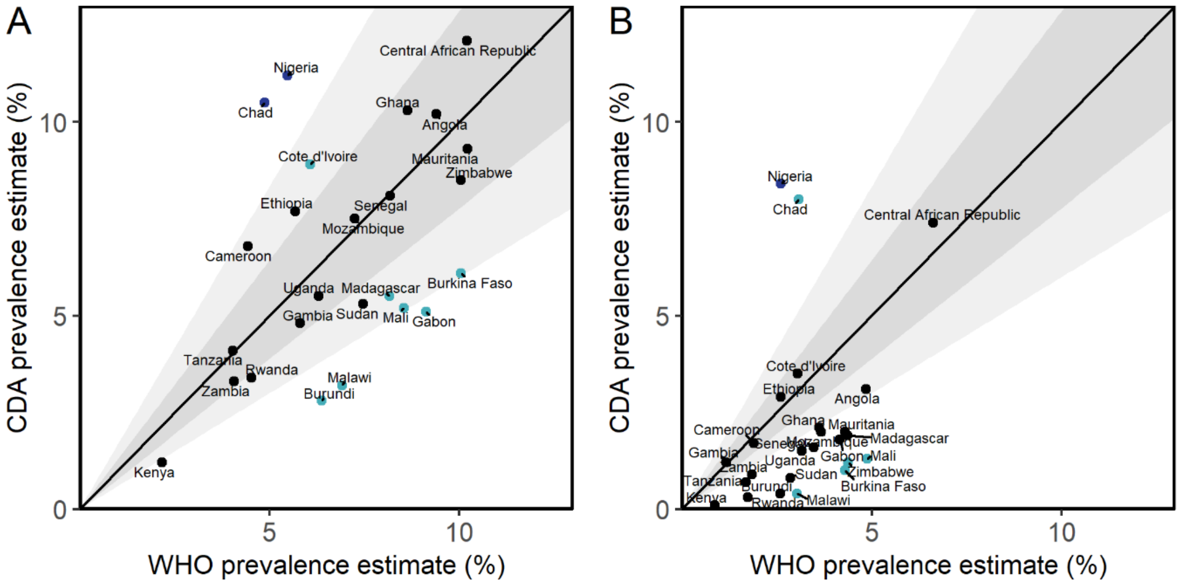

Supplementary Figure S2. Comparison of estimates from the World Health Organization (WHO) and CDA Foundation (CDA) in sub-Saharan Africa for chronic hepatitis B virus infection prevalence (A) across all ages and (B) in children under 5 years of age. Estimates for the 25 countries covered by both groups are shown. Identical estimates fall on the diagonal black line, and dark and light grey areas show relative differences between estimates of up to 25% and 50%, respectively. Countries for which estimates differ by more than 2.5 and 5 percentage points are highlighted in light and dark blue, respectively.

Supplementary Table S2. Comparison of CDA Foundation (CDA) and World Health Organization (WHO) input data sources for the 11 countries with the largest pairwise relative differences between estimates of chronic hepatitis B virus infection prevalence across all ages. Studies are cited in Polaris Observatory Collaborators, 2018, *The Lancet Gastroenterology & Hepatology* (Supplementary Appendix Table 5) and on www.whohbsagdashboard.com.

	CDA		WHO				Pairwise difference	
**Country**	**Study details**	**Reported score[a]**	**Study numbers and details**	**Publication dates**	**Total sample size**	**Including CDA study?**	**Absolute (percentage points)**	**Relative (%)**
**Nigeria**	Nationwide community-based serosurvey (2016)	2	78 studies, nearly all conducted on sub-national level and many in specific population groups (mainly pregnant women)	1970-2016	58683	No	5.7	69
**Chad**	Sub-national study in attendants of HIV testing center (2014)	3	0 studies	/	0	No	5.6	73
**Burkina Faso**	Preliminary results from unpublished general population study (2016)	3	7 studies, in pregnant women and blood donors	2001-2012	70258	No	4	49
**Gabon**	Sub-national community-based study (mixed urban and rural population) (2009)	3	7 studies, sub-national community-based (urban, rural or both)	1988-2009	5648	Yes	4	57
**Malawi**	National study in diverse participants of previous unrelated research studies (2015)	1	3 sub-national studies, in pregnant women and male occupational population	1998-2015	1183	Yes	3.7	73
**Burundi**	Nationwide community-based serosurvey (2013)	3	2 sub-national studies, in an urban and a rural population	1990-1997	219	No	3.6	78
**Mali**	Sub-national community-based study in rural Mali (1981)	2	4 studies, in pregnant women and women of childbearing age	1990-2012	5873	No	3.3	49
**Côte d'Ivoire**	Sub-national study in firefighters (2011)	2	7 studies, including 1 older national study and 4 in specific population groups (e.g. women of childbearing age and attendants of HIV testing center)	1990-2011	8108	Yes	2.8	38
**Madagascar**	National community-based serosurvey (2017)	3	6 studies, of which 4 sub-national community-based and 2 in pregnant women, blood donors and hospital outpatients	1986-2015	4027	No	2.7	39
**Cameroon**	Unpublished national community-based serosurvey (2015)	3	22 studies, all in specific population groups (pregnant women, blood	1985-2016	29371	No	2.4	42

Country	CDA Study details	Reported score[a]	WHO Study numbers and details	Publication dates	Total sample size	Including CDA study?	Pairwise difference Absolute (percentage points)	Relative (%)
			donors, ethnic groups, healthcare workers) or schoolchildren					
**Kenya**	National community-based survey (2016)	3	8 studies, including at least 3 in the general population and the study chosen by the CDA; the others in hospital outpatients and pregnant women	1977-2016	11477	Yes	0.97	58

[a] 1 = estimate based on expert opinion (default), 2 = estimate based on published or unpublished data, 3 = estimate based on a well-designed national study, unpublished or ahead of print, or a large national database.
